# The range of confidence scales does not affect the relationship between confidence and accuracy in recognition memory

**DOI:** 10.1186/s41235-017-0086-z

**Published:** 2017-12-20

**Authors:** Eylul Tekin, Henry L. Roediger

**Affiliations:** 1 Psychology Building, Campus Box 1125, One Brookings Drive, St. Louis, MO 63130 USA; 2 Washington University in St. Louis, One Brookings Drive, St. Louis, MO 63130-4899 USA

**Keywords:** Confidence-accuracy relationship, Confidence scales, Metacognition

## Abstract

Researchers use a wide range of confidence scales when measuring the relationship between confidence and accuracy in reports from memory, with the highest number usually representing the greatest confidence (e.g., 4-point, 20-point, and 100-point scales). The assumption seems to be that the range of the scale has little bearing on the confidence-accuracy relationship. In two old/new recognition experiments, we directly investigated this assumption using word lists (Experiment 1) and faces (Experiment 2) by employing 4-, 5-, 20-, and 100-point scales. Using confidence-accuracy characteristic (CAC) plots, we asked whether confidence ratings would yield similar CAC plots, indicating comparability in use of the scales. For the comparisons, we divided 100-point and 20-point scales into bins of either four or five and asked, for example, whether confidence ratings of 4, 16–20, and 76–100 would yield similar values. The results show that, for both types of material, the different scales yield similar CAC plots. Notably, when subjects express high confidence, regardless of which scale they use, they are likely to be very accurate (even though they studied 100 words and 50 faces in each list in 2 experiments). The scales seem convertible from one to the other, and choice of scale range probably does not affect research into the relationship between confidence and accuracy. High confidence indicates high accuracy in recognition in the present experiments.

## Significance

Confidence ratings are collected routinely in many types of research, including psychophysics and perception, decision making, recognition memory, eyewitness memory, and many metacognition experiments. Outside the laboratory, confidence is measured in settings such as eyewitness identification and surveys for consumer products, among others. A wide variety of confidence scales are used, ranging from simple 2-point scales (sure-unsure) to increasingly fine-grained scales ranging up to 100-point scales (where 100 is the highest confidence and 1 is guessing). Very little evidence exists to answer the question whether certain types of confidence scale are better than other types of scales. We report two recognition memory experiments using words and faces as the study materials, and we show that four scales that varied over a wide range of values (1–4, 1–5, 1–20, and 1–100) are generally comparable in their sensitivity in recognition decisions. This outcome will be reassuring to anyone who uses confidence scales. In addition, we obtained a very strong relationship between confidence and accuracy in our experiments—about as high as in eyewitness experiments—even though we had subjects study many words or faces. As in eyewitness experiments with a single tested face, our studies show that high confidence indicates high accuracy, even in experiments with many events to be remembered.

## Background

Psychologists have long wrestled with the issue of how confidence and accuracy of memories are related. The first experiment we can find asking (and answering) this question was published over 100 years ago. Dallenbach (1913) showed “observers” (using the terminology of the day) complex pictures for 1 minute each with instructions to remember them. He later tested them 5, 15, or 45 days later. One test involved asking his subjects questions about the pictures; if they provided an answer, he asked them to rate their confidence on a 3-point scale defined verbally as “slightly sure, fairly certain, or absolutely certain.” Dallenbach showed that forgetting occurred over time, which is no surprise, and he also found that confidence of responses was related to their accuracy. He concluded, “The degree of certainty of the observer’s replies bears a direct relation to the fidelity of the answer” (p. 335).

The question posed by Dallenbach in 1913 has been addressed in hundreds of experiments in the intervening century, and the relationship can be examined in many different ways, such as across subjects (Are subjects who are highly confident also highly accurate?), across events or items (Are events that are accurately remembered also confidently remembered?), within individuals (the relationship between confidence and accuracy for different events for the same person), among others (*see* Roediger, Wixted, & DeSoto, 2012, for a review). Depending on the way the question is posed and the type of analysis used, researchers have obtained every imaginable answer: strong positive correlations between confidence and accuracy, null relationships, and even negative correlations (e.g., DeSoto & Roediger, 2014; Koriat, 2008; Sampaio & Brewer, 2009). Despite the array of findings in the literature, the field is making good progress in understanding confidence-accuracy relationships in memory. Several reviews provide emerging principles that help resolve the confidence-accuracy puzzle (Koriat, 2012; Roediger & DeSoto, 2015; Wixted, Mickes, Clark, Gronlund, & Roediger, 2015; Wixted & Wells, 2017).

The aim of the present experiments was to examine a neglected factor in considering confidence-accuracy relationships: the range of the confidence scale. In reviewing the various literatures on confidence and accuracy, we found that the type of confidence scale used varies tremendously, and rarely does a researcher defend the confidence scale used (and then the defense amounts mostly to a personal preference). Most experiments on confidence-accuracy relationships use some form of recognition test, although, of course, analyses can be applied to recall, as in Dallenbach’s study (1913), in which he used cued recall. In recognition procedures, typically subjects view one or more events and then take a recognition test in which the studied event is mixed with unstudied events. Subjects are asked to pick the previously studied (“old”) item and then rate their confidence. In some procedures, they are also asked to rate their confidence in items they call unstudied (“new”). Confidence scales can range anywhere from 2 points (subjects using yes/no or old/new represents a 2-point scale), or, after making a yes/old judgment, researchers have used 3-point scales (Dallenbach, 1913), 5-point scales (Read, Yuille, & Tollestrup, 1992), 6-point scales (Perfect, 2004), 7-point scales (Brewer & Sampaio, 2012), 9-point scales (Robinson & Johnson, 1996), 20-point scales (Mickes, Hwe, Wais, & Wixted, 2011), and 100-point scales (DeSoto & Roediger, 2014). As noted, the general assumption seems to be that various scales are used in much the same way, because few researchers bother to tell why they used a particular scale or include two or more scales in their research to examine whether their findings are generalizable across scale types. We examined the issue directly in two experiments, and we review the evidence that is already available on the issue of how the type of scale may affect the relationship between confidence and accuracy.

Previous research on decision making in recognition memory addressed whether more decision options led to greater decision noise. Malmberg and Xu (2006) used a 4-point recognition scale (4 points from “sure yes” at 4 to “sure no” at 1) and Benjamin, Diaz, and Wee (2009) manipulated the set size of options in the recognition test by presenting one, two, or four words in each set. They defined accuracy as discriminability and calculated discrimination of targets from lures using ROCs. The researchers in both of these studies concluded that the ROC functions were influenced not just by stimulus noise (as they should be) but also by decision noise; as the number of decision options increased, the recognition measures became less trustworthy.

To directly test this claim, Benjamin, Tullis, and Lee (2013) conducted a recognition experiment with words and manipulated the range of the scale for the recognition decision between subjects. Subjects provided recognition judgments using only two-value (i.e., binary yes/no) or four- or eight-value scales. On the four- and eight-value scales, the lowest value was labeled “sure no,” whereas the highest value was labeled “sure yes.” Benjamin et al. concluded that the more alternatives given, the poorer the performance: “Rating scales with more options led to lower estimates of recognition than did scales with fewer options” (p. 1601) (but see Kellen, Klauer, & Singmann, 2012). However, one important difference between the procedure in this experiment and that in most confidence-accuracy research is that, in the latter research, experimenters first asked subjects to make a binary yes/no recognition decision and then rated their confidence on a scale for that decision. Thus, in Benjamin et al.’s (2013) terms, the initial judgment is always on a binary scale. Still, this research does provide a reason to expect that in other settings subjects will not use widely varying confidence scales in the same way.

Other results suggest that scale differences in recognition memory experiments may not matter. In two recognition memory experiments, Mickes, Wixted, and Wais (2007) used 20-point or 99-point rating scales to assess confidence for all items. The idea behind switching from a 20-point scale to a 99-point scale was to see if subjects would use more fine-grained readings at the high end of the 99-point scale. However, for the 99-point scale, the results revealed that “subjects often supplied ratings at intervals of 5 on the scale, which means that, for them, this was effectively a 20-point scale” (p. 863). Even though the comparison of 99-point and 20-point scales was not the main purpose of their study, Mickes et al. (2007) showed that 20- and 99-point scales yielded similar confidence-accuracy distributions. Of course, both these scales are relatively large, and many researchers use narrower scales (e.g., 1–4), so one can wonder if the conclusion would hold over a wider variety of scales.

More directly relevant to our present project, Dodson and Dobolyi (2015) compared nine confidence scales using lineup identifications as recognition tests. They employed verbal and numeric scales (e.g., ranged from 0 to 100 or from “not at all confident” to “completely confident”) and different numbers of points identified on a 100-point scale (e.g., numeric 6 points, 0–100: 0, 20, 40, 60, 80, or 100). They also manipulated whether the 100-point scale started at 0 or 50 (e.g., numeric 6 points, 50–100: 50, 60, 70, 80, 90, or 100) and whether they gave labels only for end points on verbal scales (e.g., using 6 points but only with “not at all confident” and “completely confident” labels on the end points). Thus, for verbal scales, they had 6 points with each point labeled, 11 points with each one labeled, 6 points with only the end points labeled, and 11 points with only the end points labeled. For numeric scales, they had 6 points with 0–100, 6 points with 50–100, 11 points with 0–100, and 11 points with 50–100. They also used a continuous numeric scale ranging from 0 to 100 with a slider, and thus overall they used nine different confidence scales. They analyzed the results derived from these scales in using confidence-accuracy calibration measures as well as correlational measures. They showed that the confidence-accuracy relationship was generally the same with all types of scales. Of course, in some sense, all their measures were variations on using a 100-point confidence scale.

The prior research is a bit mixed on the question whether various confidence scales provide the same estimate of the relationship between confidence and accuracy. Our experiments address this same issue, but in a different manner from past research. We compared subjects’ use of 4-, 5-, 20-, and 100-point scales in recognizing words (Experiment 1) and faces (Experiment 2) using confidence-accuracy characteristic (CAC) plots (Mickes, 2015). These plots permit us to ask questions such as, “Is 5 on a 5-point scale equivalent to 17–20 on a 20-point scale and to 81–100 on a 100-point scale in terms of accuracy?” Of course, we can ask this question for all points on the confidence scale (“Is a 2 on a 4-point scale equivalent to 6–10 ratings on a 20-point scale and 26–50 on a 100-point scale?”). One essential difference between the present study and that of Dodson and Dobolyi (2015) is that we used confidence scales over a wide range (4-, 5-, 20-, and 100-point scales) rather than carving up a 100-point scale in different ways. At issue is whether subjects will use these widely different confidence scales in the same way or in different ways. This issue is of practical significance because both researchers and police departments want to use the most sensitive type of scale.

The present experiments addressed three primary questions: First, do different ranges of confidence scales yield similar confidence-accuracy relationships? Second, do the highest points of each scale yield similar accuracy rates? The reasoning behind the second question was that the highest point on confidence scales with more points (i.e., 20- and 100-point scales) may provide higher accuracy than confidence scales with fewer points (i.e., 4- and 5-point scales). Third, what do CAC plots reveal for experiments in which many items are used (100 words in our first experiment and 50 faces in our second experiment)? CAC plots have thus far been employed only in eyewitness identification experiments, which are almost always one-item (one crime and lineup) experiments. CAC plots in these eyewitness experiments show that, on an initial identification from a lineup, high confidence always indicates high accuracy (Wixted et al., 2015; Wixted & Wells, 2017). However, this outcome may break down when large numbers of targets are used, owing to interference among items. However, the nature of CAC plots in experiments with many words or faces is an empirical issue that the present experiments help to resolve.

## Experiment 1

In Experiment 1, subjects sequentially studied two different sets of 100 words and were tested on 200 words (100 targets, 100 lures) after each study phase. The lures were primary associates of the targets to make the tests difficult. After each old/new decision, different groups of subjects gave confidence judgments using a 4-, 5-, 20-, or 100-point scale.

### Methods

#### Subjects

Subjects were 96 Washington University undergraduate students who participated for payment or course credit in groups of one to five. Data from two subjects were lost because of a programming error, and these subjects were replaced. Subjects were randomly assigned to one of the four confidence scales, with 24 subjects in each condition. The study was approved by the Washington University Institutional Review Board.

#### Design and materials

The experiment used a between-subjects design that manipulated only one variable: the type of confidence scale used on the yes/no recognition test. Four different confidence scales were used, and the recognition tests differed only in terms of the range of the confidence scale. After subjects judged a test item to be old or new, they rated their confidence on a scale of 1–4, 1–5, 1–20, or 1–100, with labels at each end of the scale ranging from “not confident at all” on the low end to “totally confident” on the high end. Thus, four groups of subjects were tested.

Word sets were used as materials for the present experiment. Two hundred associated word pairs (thus 400 words) were selected from among the Nelson, McEvoy, and Schreiber (2004) norms, with all associated items being one of three strongest associations of the target word (e.g., *table–chair*). The words had concreteness levels greater than 3.5 of 7 according to Nelson et al. (2004). The logarithm of HAL frequency in the English Lexicon Project (Balota et al., 2007) was used as a measure to check for word frequencies, which ranged from 5.98 to 13.67. The two items were counterbalanced across study and test phases. For example, for half of the subjects, when *table* was presented during the study phase, *chair* served as the lure during the test phase; for the other half, *chair* served as the target and *table* as the lure. Thus, all 400 words appeared as both targets and lures across subjects. Each study list consisted of 100 words presented in random order (different for each subject), and the recognition test consisted of 200 words (targets and their primary associates), also presented in random order.

Two filler tasks, a president recognition test (Roediger & DeSoto, 2016) and a survey about the events in Ferguson, Missouri, in 2015, were used in the experiment between study of each list and its test. The filler tasks were counterbalanced across the first and second lists. The tasks are tests used in other research in our laboratory and permit an assessment of undergraduate knowledge of presidents and the events surrounding Michael Brown’s death in Ferguson. These tasks were selected because they should provide general, not specific, interference in remembering lists of words.

#### Procedure

After subjects were given a consent form that included general information about the experiment, they were told they would be presented with words one at a time and be asked to remember them for a later memory test. The experiment consisted of two halves, and each half had three phases: study of the list, a distractor task, and a recognition test. During the study phase, 100 words were presented in the middle of the computer screen for 2 seconds each, with a 500-millisecond blank screen between words, for an effective study duration of 2.5 seconds. After the study phase, subjects completed one of the 10-minute filler tasks described above. During the recognition phase, 200 words (100 previously studied words and 100 related lures) were presented one at a time to the subjects. For each word, subjects responded whether they had seen the word during the study phase by clicking “old” or “new” on the screen. After making this decision, they were asked to make a confidence judgment about their answer on the given scale. They were informed that the highest point on the scale indicated “totally confident” and the lowest point indicated “not confident at all.”

Subjects rated confidence on 4-point, 5-point, 20-point, and 100-point scales (ranging from 1 to the highest point of the given scale). We selected these scales so that they would be easily converted to one another for comparison. That is, both 20-point and 100-point scales can be divided into four and five bins to be compared with 4-point and 5-point scales. The recognition test was self-paced, and subjects typed in a number (1–4, 1–5, 1–20, or 1–100) to indicate confidence. They were required to make a confidence judgment before moving to the next test item. After completing this procedure for 200 words, subjects took a 5-minute break and then started the second study phase with a different set of 100 words. Other than the new set of material and the alternative filler task, other aspects of the procedure were the same as in the first half of the experiment. After the subjects completed the second round, they were debriefed. The experiment lasted for 60–90 minutes, depending on subjects’ pace of responding.

### Results

The top section of Table 1 provides the hit rates, false alarm rates, and *d′* for the four different rating scale conditions. To examine whether hit and false alarm rates differed between the first and second phases of the experiment, we conducted two separate 2 (phase 1 vs. phase 2) × 4 (scales) analyses of variance (ANOVAs) for hit and false alarm rates. For both hit rates and false alarm rates, the results revealed that phases and the type of scale did not differ on these dimensions; for hits, *F*(1,92) = .82, *BF*_*01*_ = 6.35, *F*(3,92) = 1.06, *BF*_*01*_ > 100, and for false alarms, *F*(1,92) = 1.53, *BF*_*01*_ = 4.41, *F*(3,92) = 1.70, *BF*_*01*_ = 70.60, respectively (*p*_s_ > .05). Hence, Table 1 presents the data collapsed across the two phases, and we used these combined data for all analyses. For *d′* scores, one-way between-subjects ANOVA revealed no main effect of the type of scale: *F*(3,92) = .60, *p* = .619, η^2^_p_ = .02, *BF*_*01*_ > 100.

**Table 1:** Hit rates, false alarm rates and sensitivity scores for Experiments 1 and 2

Scale type	Hits		False alarms		*d′*	
	Mean	SD	Mean	SD	Mean	SD
Experiment 1						
100-point scale	.69	.13	.37	.16	.89	.60
20-point scale	.71	.14	.36	.14	.99	.73
5-point scale	.65	.17	.28	.15	1.08	.70
4-point scale	.72	.15	.33	.19	1.17	.95
Experiment 2						
100-point scale	.74	.11	.17	.13	1.77	.69
20-point scale	.68	.12	.15	.09	1.64	.56
5-point scale	.70	.13	.19	.12	1.50	.70
4-point scale	.71	.14	.16	.08	1.66	.60

#### Comparison of hits across confidence scales

For each bin, accuracy is computed by using the following formula: Proportion correct = number of hits/(number of hits + number of false alarms). To investigate the relationship of accuracy across the groups using the four scales, we analyzed the data by converting the 20- and 100-point scales into bins that permitted comparison. We used four bins for the 4-point scale and five bins for the 5-point scale. That is, for comparison with the 4-point scale, we binned data from subjects using the 20-point scale into bins that contained the number of responses made from 1–5, 6–10, 11–15, and 16–20 on the scale. Similarly, for the 100-point scale, we binned the data into bins of 1–25, 26–50, 51–75, and 76–100. We used the same analytic approach for the 20- and 100-point data for comparison with the 5-point scale. With this analysis, for example, we compared accuracy at the 5-point confidence level on a 5-point scale with 81–100 and 17–20 ranges on 100- and 20-point scales, respectively. Subjects used ratings in the lower confidence bins relatively rarely, so fewer observations were obtained in these bins. Therefore, the lowest two confidence bins were combined for further analyses. The number of observations per confidence bin is provided in Appendix 1.

**Fig. 1:**
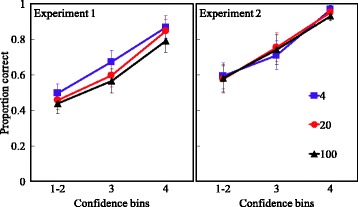
Comparison of hits across three confidence bins for 4-, 20-, and 100-point scales for Experiment 1 (*left panel*) and Experiment 2 (*right panel*). The first bin in both cases combines ratings of 1 and 2 to increase the number of observations at the low confidence part of the scale. All points are calculated using the following formula: number of hits in a confidence bin/(number of hits + number of false alarms in that confidence bin). Error bars indicate 95% CI

**Fig. 2:**
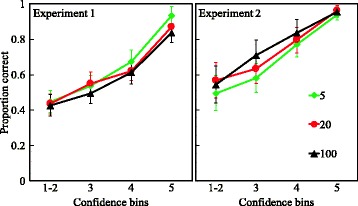
Comparison of hits across four confidence bins for 5-, 20-, and 100-point scales for Experiment 1 (*left panel*) and Experiment 2 (*right panel*). The first bin in both cases combines ratings of 1 and 2 to increase the number of observations at the low confidence part of the scale. All points are calculated using the following formula: number of hits in a confidence bin/(number of hits + number of false alarms in that confidence bin). Error bars indicate 95% CI

Figs. 1 and 2 show these comparisons for four confidence bins and five confidence bins, respectively, for hits. As shown in both figures, accuracy increased steadily as a function of confidence, and the scale type did not lead to any difference in the increased accuracy with confidence. In Fig. 1 (*left panel*), mean accuracy ratios for the bins from 1–2 to 4 were .46, .61, and .83. For the 5-point scale, the corresponding values were .44, .53, .64, and .88 (Fig. 2, *left panel*). Obviously, if subjects are more confident, they are also more accurate. This outcome occurred despite our making the recognition test difficult by using primary associates as lures.

Two two-way repeated-measures ANOVAs were conducted, with confidence bins serving as the within-subjects factor and type of rating scale as the between-subjects factor. First, for the comparison of 100-, 20-, and 4-point scales, a 3 (confidence bins) × 3 (scales) ANOVA was conducted, which revealed a main effect of confidence bins, *F*(1.77,122.18) = 147.00, *p* < .001, η^2^_p_ = .68, and a main effect of the type of scale, *F*(2,69) = 3.41, *p* = .039, η^2^_p_ = .09, but no interaction *F*(3.54,122.18) = .41, *p* = .778, η^2^_p_ = .01. The pairwise comparisons with the Šidák correction revealed that, overall, the 4-point group (mean .68, SE.02) showed higher accuracy than the 100-point group (mean .60, SE.02), *p* = .033. Second, a 4 (confidence bins) × 3 (scales) ANOVA was conducted for comparison of the 100-, 20-, and 5-point scales, revealing a main effect of confidence bins, *F*(2.27,156.83) = 167.29, *p* < .001, η^2^_p_ = .71, but no main effect of type of scale, *F*(2,69) = 1.87, *p* = .162, η^2^_p_ = .05, *BF*_*01*_ = 10.72. The interaction was not reliable, *F*(4.55, 156.83) = .71, *p* = .601, η^2^_p_ = .02. The results of Experiment 1 revealed that higher confidence led to higher accuracy. In addition, subjects using the 100-point scale were less accurate than subjects using the 4-point scale. This is interesting because the two groups did not differ in their overall hit and false alarm rates. Moreover, this pattern did not emerge for 5-point comparisons. We examined this issue again in Experiment 2.

#### Comparison of hits at the most confident point of each scale

The previous analysis showed no consistent pattern for points at the highest range of confidence (i.e., bin 4 or 5, depending on the range of the scales). However, perhaps differences would appear if we had considered only the highest possible point in each scale type, such as the proportion correct for ratings of 4, of 5, of 20, and of 100 for the four different scale types. We hypothesized that accuracy would be highest when subjects could give 100 on a 100-point scale relative to, say, 4 on a 4-point scale, owing to the finer grain of the 100-point scale. Hence, we compared proportion correct for the last points of each scale; thus, for the 100- and 20-point scales, hits arising from only the 100- and 20-point ratings were included in the comparison. The logic behind the comparison was that in wide-range scales, the highest point at the highest end of confidence (e.g., 100 at the 81–100 bin) might yield higher accuracy than the highest point in narrow-range scales (e.g., 4 or 5 points). The number of ratings for the most confident response (4, 5, 20, or 100) sharply decreased from 4-point scales to 100-point scales (*see* Appendix 2). Still, we can ask if accuracy increased across scales at the most confident point, and the logic above leads to the prediction that accuracy should be higher for subjects using 20- and 100-point scales.

The mean proportions correct for the highest confidence rating were as follows: for ratings of 4 (mean .87, SD .16), of 5 (mean .93, SD.10), of 20 (mean .92, SD .13), and of 100 (mean .94, SD .11). A one-way between-subjects ANOVA was conducted across the four scale conditions and revealed no effect of scale type, *F*(3,90) = 1.46, *p* = .230, η^2^_p_ = .05, *BF*_*01*_ = 97.24, which surprised us, given the much larger numbers of observations in the four and five bins for the more coarse grain scales (4 and 5; *see* Appendix 2).

#### Comparison of correct rejections across confidence scales

When subjects correctly rejected an unstudied item by picking “new,” they also made a confidence judgment on this correct response. Thus, we can also assess the relationship between confidence and accuracy for correct rejections using CAC plots. We first examined whether the groups differed from one another in correct rejection rates through one-way between-subjects ANOVA, and no difference was found among groups, *F*(3,92) = 1.68, *p* = .173, η^2^_p_ = .05. Correct rejection rates for the 4-, 5-, 20-, and 100-point confidence scales were .67, .73, .64, and .63, respectively. Then, for each bin, accuracy was computed by using the following formula: proportion correct = number of correct rejections/(number of correct rejections + number of misses). As with analyses of hits, we combined the lowest two confidence bins because of the low number of observations. The number of observations per bin is provided in Appendix 3.

**Fig. 3:**
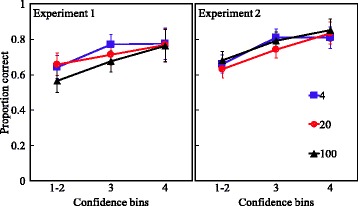
Comparison of correct rejections across four confidence bins for 4-, 20-, and 100-point scales for Experiment 1 (*left panel*) and Experiment 2 (*right panel*). The first bin in both cases combines ratings of 1 and 2 to increase the number of observations at the low confidence part of the scale. All points are calculated using the following formula: number of correct rejections in a confidence bin/(number of correct rejections + number of misses in that confidence bin). Error bars indicate 95% CI

**Fig. 4:**
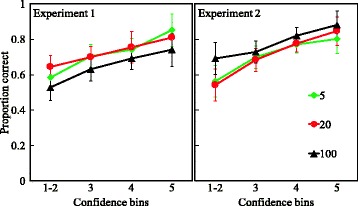
Comparison of correct rejections across four confidence bins for 5-, 20-, and 100-point scales for Experiment 1 (*left panel*) and Experiment 2 (*right panel*). The first bin in both cases combines ratings of 1 and 2 to increase the number of observations at the low confidence part of the scale. All points are calculated using the following formula: number of correct rejections in a confidence bin/(number of correct rejections + number of misses in that confidence bin). Error bars indicate 95% CI

We investigated the relationship between correct rejections and confidence in the same way we investigated the relationship between confidence and hits, dividing 100-point and 20-point scales into bins of five or four. Figs. 3 and 4 (*left panels*) show that probability of correct rejections increased with increasing confidence, and the scale type did not create much difference in terms of correct rejections. For the comparison of 100-, 20-, and 4-point scales for the data in Fig. 3 (*left panel*), a 3 (confidence bins) × 3 (scales) ANOVA again revealed a main effect of confidence bins, *F*(1.51,102.39) = 28.13, *p* < .001, η^2^_p_ = .29, but no effect of scale type, *F*(2,68) = 1.11, *p* = .337, η^2^_p_ = .03, *BF*_*01*_ = 22.72, and no interaction *F*(3.01,102.39) = 1.50, *p* = .220, η^2^_p_ = .04.

For the data in Fig. 4 (*left panel*), a 4 (confidence bins) × 3 (scales) ANOVA for the comparison of 100-, 20-, and 5-point scales indicated a main effect of confidence bins, *F*(2.05,129.40) = 37.42, *p* < .001, η^2^_p_ = .37, but no main effect of the type of scale, *F*(2,63) = 2.18, *p* = .122, η^2^_p_ = .07, *BF*_*01*_ = 7.25, with no reliable interaction, *F*(4.11,129.40) = .88, *p* = .481, η^2^_p_ = .03.

#### Comparison of correct rejections at last point of each scale

We compared the accuracy of correct rejections for the last point of each scale as we did with hits. A one-way between-subjects ANOVA was conducted between 100 (mean .82, SD .29), 20 (mean .84, SD .25), 5 (mean .85, SD .19), and 4 (mean .77, SD .25) points, which revealed no main effect of scale type, indicating that accuracy for correct rejections at the highest point did not differ from one another as a function of scale type, *F*(3,70) = .44, *p* = .727, η^2^_p_ = .02, *BF*_*01*_ > 100.

#### Comparison of confidence-accuracy relationship between hits and correct rejections

**Fig. 5:**
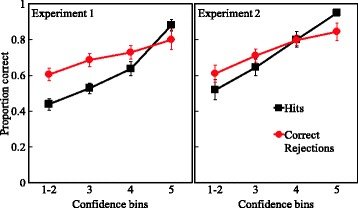
Comparison of hits and correct rejections across confidence bins for Experiment 1 (*left panel*, *n* = 67) and Experiment 2 (*right panel*, *n* = 56). The reduction in number of subjects is due to not all subjects contributing to lowest ratings. Data are collapsed across 5-, 20-, and 100-point scales. The first bin in both cases combines ratings of 1 and 2 to increase the number of observations at the low confidence part of the scale. Error bars indicate 95% CI

A comparison of the data in Figs. 1 and 2 (hits) with data in Figs. 3 and 4 (correct rejections) indicates that the confidence-accuracy relationship appears steeper for hits than for correct rejections. The data are shown in Fig. 5 (*left panel*) for the 5-point scale, thus collapsing across the data in the *left panels* of Figs. 2 (hits) and 4 (correct rejections). We conducted a 2 (hits, correct rejections) × 4 (confidence bins) ANOVA and obtained a main effect of level of confidence, *F*(2.17,136.49) = 153.37, *p* < .001, η^2^_p_ = .71, and a reliable interaction, *F*(2.51,158.18) = 39.51, *p* < .001, η^2^_p_ = .39. Overall, the proportion correct for correct rejections (mean .70, SE .02) was higher than the proportion correct for hits (mean .62, SE .01), *F*(1,63) = 29.44, *p* < .001, η^2^_p_ = .32. The post hoc pairwise comparisons, though, revealed that the interaction was driven by a crossover between hits and correct rejections at the highest end of the confidence scales. The proportions of correct rejections were higher than proportions of hits at the first (mean .61, SE .02, mean .44, SE .02), second (mean .69, SE .02, mean .53, SE .02), and third bins (mean .73, SE .02, mean .64, SE .02), *p*_s_ < .001. Yet, at the fifth bin, the proportion of hits (mean .88, SE .02) was higher than the proportion of correct rejections (mean .80, SE .03, *p* < .001). Hence, the confidence-accuracy relationship for hits is indeed steeper than that for correct rejections. The same pattern occurred for the 4-point confidence scale.

Before discussing the results, we attempted to replicate them using faces as the study material with the same basic design as in Experiment 1.

## Experiment 2

In this experiment, we switched to faces as the to-be-remembered material, because previous literature suggested that confidence and accuracy might change according to the type of material (Roediger et al., 2012). This might be one reason for the differences observed in the confidence-accuracy relationship between list-learning and eyewitness situations. Thus, we aimed to replicate (or not) the findings from Experiment 1 with faces. Would the various confidence scales be used similarly with faces as they are with words?

### Methods

#### Subjects

The subjects were 97 undergraduate students from Washington University’s psychology subject pool, and they participated for either payment or course credit in groups of one to five. One of the subjects was sleeping during the experiment and was replaced. Subjects were randomly assigned to one of the four confidence scales, with 24 subjects in each condition.

#### Design and materials

In Experiment 2, 200 neutral faces were selected from Minear and Park’s (2004) face database as materials. The face set consisted of 100 females and 100 males, their ages ranging from 19 to 50 years; 80% of faces were Caucasian Americans, and the remaining 20% were African Americans. These percentages were distributed equally between genders. Similarly to Experiment 1, when a particular face was presented as a target for half of the subjects, the same face served as a lure during a recognition test for the remaining subjects. Thus, all 200 faces appeared as both targets and lures across subjects.

The same two filler tasks and the same type of yes/no recognition test with 4-point, 5-point, 20-point, and 100-point confidence scales from Experiment 1 were used. In Experiment 2, we also employed a between-subjects design with four different confidence scales and 24 subjects per condition.

#### Procedure

Similarly to the procedure of Experiment 1, Experiment 2 had two study/test phases. Subjects were presented with 50 faces for 2 seconds each with a 500-millisecond blank screen between faces. After this study phase, subjects worked on a 10-minute filler task and then started the first recognition test, which consisted of 100 faces (50 previously studied faces and 50 lures). Besides the different material, the recognition test had the same structure and instructions as in Experiment 1. The second half of the experiment started after the 5-minute break after the first test with a different set of 100 faces, 50 studied and 100 tested. Faces were randomly presented during both study and test phases, with a different randomization for each subject. The whole procedure lasted approximately 60 minutes, depending on subject’s pace.

### Results

As in Experiment 1, the bottom section of Table 1 shows the overall performance of the four scale groups. To examine whether the first and second phases of the experiment differed, we again conducted two 2 (phase 1 vs. phase 2) × 4 (scales) ANOVAs for hit rates and false alarm rates. For hit rates, phase revealed a significant main effect, *F*(1,92) = 8.99, *p* = .003, η^2^_p_ = .09, with phase 2 (mean .73, SE .02) leading to more hits than phase 1 (mean .69, SE .02). The type of scale did not differ, *F*(3,92) = .95, *BF*_*01*_ > 100, and the interaction was not reliable, *F*(3,92) = .43 (*p*_s_ > .05). For false alarm rates, the results revealed that phases and the type of scale did not differ, *F*(1,92) = 2.51, *BF*_*01*_ = 2.72, *F*(3,92) = .74, *BF*_*01*_ > 100, respectively (*p*_s_ > .05). In addition, for *d′* scores, a one-way between-subjects ANOVA revealed no main effect of the type of scale, *F*(3,92) = .75, *p* = .526, η^2^_p_ = .02, *BF*_*01*_ > 100.

Table 1 presents the data collapsed across the two phases, because the face sets were counterbalanced across phases, and the difference was small and similar across all four groups. Recognition performance was clearly higher for faces (bottom of Table 1) than for words (top of Table 1). This outcome might be due to the difference in materials (words and faces), the number of studied items (200 or 100), or the nature of the lures (primary associates of the words in Experiment 1 but with no similar deliberate manipulation in Experiment 2). The data in Table 1 led us to suspect that the nature of lures made the difference, because the false alarm rates for words in Experiment 1 were much higher than those for faces in Experiment 2. Whatever the reason, Experiment 2 permitted us to ask if the conclusions drawn from Experiment 1 replicated with faces and with more accurate recognition performance.

As in Experiment 1, the lowest two confidence bins were combined across different confidence levels for further analyses, owing to the relative paucity of observations at the lower ends of the confidence scale. The numbers of observations are shown in Appendix 1 for hits and in Appendix 3 for correct rejections.

#### Comparison of hits across confidence scales

As in Experiment 1, the 100- and 20-point scales were again divided into either four bins (to compare with the 4-point scale) or into five bins to compare with the 5-point scale. Figs. 1 and 2 (*right panels*) show these comparisons for four confidence bins and five confidence bins, respectively. For the 4-point scale, mean accuracy for the bins was 1–2 to 4 was .58, .74, and .95, respectively, and for 5-point scale, the corresponding values were .53, .64, .80, and .95.

Two separate two-way repeated-measures ANOVAs were conducted, with confidence bins serving as the within-subjects factor and scales serving as the between-subjects factor. For the data in Fig. 1 (*right panel*), a 3 (confidence bins) × 3 (scales) ANOVA indicated a main effect of confidence bins, *F*(2,136) = 154.13, *p* < .001, η^2^_p_ = .69, no main effect of scale type, *F*(2,68) = .03, *p* = .968, η^2^_p_ = .001, *BF*_*01*_ = 68.55, and no reliable interaction, *F*(4,136) = .70, *p* = .594, η^2^_p_ = .02. Second, for the data displayed in Fig. 2 (*right panel*), a 4 (confidence bins) × 3 (scales) ANOVA revealed a main effect of confidence bins, *F*(2.11,136.98) = 92.85, *p* < .001, η^2^_p_ = .59, no main effect of scale type, *F*(2,65) = 1.71, *p* = .189, η^2^_p_ = .05, *BF*_*01*_ = 11.86, and no reliable interaction *F*(4.22, 136.98) = .76, *p* = .563, η^2^_p_ = .02. Thus, in Experiment 2, in both analyses, no difference appeared at the confidence bins from subjects using scales of different ranges, from a 4-point scale to a 100-point scale. The results generally replicated those of Experiment 1, and the observed difference between 4-point and 100-point scales did not emerge in Experiment 2. Subjects seemed to scale their confidence appropriately in using the widely different scale ranges. Accuracy was strongly affected by increasing levels of confidence; however, accuracy did not differ among confidence scales at the higher confidence levels with the most observations.

#### Comparison of hits at the most confident end of each scale

Again, we compared the proportion correct for the last point of each scale. A one-way between-subjects ANOVA was conducted for 100 (mean .98, SD .04), 20 (mean .98, SD .02), 5 (mean .94, SD .09), and 4 points (mean .97, SD .05), and it revealed a significant effect for scale type, *F*(3,88) = 2.98, *p* = .036, η^2^_p_ = .09. Pairwise comparisons revealed that the effect was driven by the marginal differences between 100-point and 5-point groups and between 20-point and 5-point groups in their respective conditions (*p* = .077, *p* = .063, respectively). True, the rating in the 5-point scales was lower than the others, but because the proportion correct for the closely comparable 4-point scale was .97 for the confidence rating of 4 and the groups were at the ceiling, the 5-point value may be artificially lower for some reason.

#### Comparison of correct rejections across confidence scales

There was no difference between groups in terms of overall correct rejection rates, *F*(3,92) = .74, *p* = .531, η^2^_p_ = .02. Correct rejections for the 4-, 5-, 20-, and 100-point confidence scales were .84, .81, .85, and .83, respectively.

As shown in Figs. 3 and 4 (*right panels*), the proportions correct for correct rejections from Experiment 2 were similar to those in Experiment 1 in showing little difference among confidence scales. A 3 (confidence bins) × 3 (scales) ANOVA indicated a main effect of confidence bins, *F*(1.75,120.86) = 45.97, *p* < .001, η^2^_p_ = .40, but no main effect of the scale type, *F*(2,69) = .96, *p* = .390, η^2^_p_ = .03, *BF*_*01*_ = 27.18, and no interaction *F*(3.50,120.86) = 1.11, *p* = .354, η^2^_p_ = .03. Second, a 4 (confidence bins) × 3 (scales) ANOVA revealed that there was a main effect of confidence bins, again confirming the relationship between confidence and correct rejections, *F*(2.34,152.18) = 32.78, *p* < .001, η^2^_p_ = .34. Pairwise comparisons revealed that the fourth confidence bin (mean .79, SD .02) and the fifth confidence bin (mean .84, SD .02) did not differ from one another in terms of accuracy (*p* = .145). There was a marginal main effect of scales, *F*(2,65) = 3.06, *p* = .054, η^2^_p_ = .09, and it was driven by the marginal difference between 5-point group (mean .71, SD .02) and the 100-point group (mean .78, SD .02), *p* = .092. The interaction was not a reliable interaction, *F*(4.68,152.18) = .95, *p* = .445, η^2^_p_ = .03. In general, these results replicate the pattern observed in Experiment 1.

#### Comparison of correct rejections at last point of each scale

Appendix 2 reports the number of observations for correct rejections at the last point for each scale. A one-way between-subjects ANOVA was conducted between 100 (mean .90, SD .15), 20 (mean .85, SD .15), 5 (mean .80, SD .29), and 4 points (mean .84, SD .13), and it revealed no main effect of scale type, *F*(3,74) = .77, *p* = .513, η^2^_p_ = .03, *BF*_*01*_ > 100, again replicating Experiment 1.

#### Comparison of confidence-accuracy relationship between hits and correct rejections

We compared the combined data in the *right panels* of Figs. 1 and 2 (hits) with those in Figs. 3 and 4 (correct rejections) to see whether the confidence-accuracy relationship between hits and correct rejections seen in Experiment 2 would replicate those in Experiment 1. Fig. 5 (*right panel*) shows the collapsed data for the 5-point confidence comparison with the lowest two bins combined. A 2 (hits, correct rejections) × 4 (confidence bins) ANOVA revealed a significant effect of level of confidence, *F*(2.65,164.11) = 141.70, *p* < .001, η^2^_p_ = .70 and a significant interaction, *F*(2.18,135.05) = 11.20, *p* < .001, η^2^_p_ = .15. Overall, the proportion correct for hits and correct rejections did not differ, *F*(1,62) = .52, *p* = .476, η^2^_p_ = .008. Pairwise comparisons revealed that in the first two confidence bins, the proportion correct for correct rejections was significantly higher than the proportion correct for hits (*p*_s_ < .05). However, as in Experiment 1, this relationship reversed at the fifth confidence bin: hits (mean .95, SE .01) were significantly higher than correct rejections (mean .84, SE .03, *p* < .001). In Experiment 2, the overall proportion correct for correct rejections was higher than the proportion correct for hits, and the confidence-accuracy relationship for hits was steeper than it was for correct rejections. Again, the same pattern held when we analyzed the other data using the four-bin data, just as in Experiment 1.

## Discussion

The two experiments we report were designed to answer three questions and did so conclusively. We review the issues and results in turn and then consider ancillary findings.

First, do confidence scales ranging from 4 and 5 points to 20 and 100 points produce different confidence-accuracy relationships? The answer is generally no. The CAC plots were remarkably similar for all scale types, especially for the middle to high ranges of confidence. This was true both for hits and for correct rejections, and the generalization also held for both word lists (Experiment 1) and sets of faces (Experiment 2). Despite the huge differences in size and grain of the scales, subjects’ behavior was quite orderly in that high confidence indicated high accuracy, with a steady drop in accuracy for less confident judgments. The type of confidence scale just does not much matter. Thus, for many practical purposes, such as in advising police departments on what type of scale to use for eyewitness identifications, the answer is that any scale will suffice. Of course, we examined only four possible scales, but ours went from grain sizes of 4 to 100. It seems unlikely that the CAC plots would not be similar for, say, 3- and 6-point scales.

CAC plots have thus far been used only for eyewitness identification experiments, which are, in the lexicon of cognitive psychologists, one-item experiments. That is, subjects view a face or a crime scene and typically attempt to identify a person from a lineup given later. The current state of the eyewitness literature (overlooking 30 years of research in which experimenters misanalyzed their data) is that high confidence indicates high accuracy when using the CAC approach, at least on an initial test (Wixted et al., 2015; Wixted & Wells, 2017). The situation is probably different on successive tests, because each assessment or test influences later tests.

Our experiments are the first to use the CAC approach to examine recognition memory for large sets of target items, and thus we can ask if the CAC plots are markedly different in recognition experiments for large numbers of events (200 words in Experiment 1 and 100 faces in Experiment 2) from in eyewitness experiments with only one target event. We cannot answer conclusively, of course, because we did not include standard eyewitness conditions in these experiments, but we can gain an impression by examining the many CAC functions shown in the recent meta-analysis by Wixted and Wells (2017). Many of the studies they reviewed used 100-point scales of confidence, so the closest comparison is with our 100-point scales, but, of course, all our scales provided similar results. In general, our results in Fig. 1 look remarkably similar to those in the eyewitness literature (*see* Fig. 5a in the paper by Wixted and Wells for an average of 15 studies, although their measure is suspect identification accuracy, and ours is proportion correct assessed as hits/hits misses in each confidence bin).

**Fig. 6:**
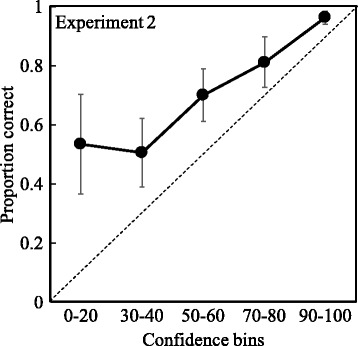
Confidence-accuracy characteristic plot for 100-point scale in Experiment 2. Points are calculated by using the following formula to resemble suspect identification accuracy: number of hits/(number of hits + number of false alarms). Confidence bins are divided to be consistent with those of Wixted and Wells (2017)

We converted our data from Experiment 2 with faces into a plot something like suspect identification accuracy in eyewitness research by computing, for each bin of 20 for the 100-point scale, hits in that bin divided by hits plus false alarms. Fig. 6 shows the graphical representation of this function. The general appearance of our function is similar to that of Wixted and Wells (2017); Fig. 5a and b, although it is difficult to compare them directly, owing to different procedures used. Still, high confidence indicates high accuracy. For example, for confidence ratings of 90–100 in our data with 100 faces, subjects are .96 accurate, whereas in their data (with one eyewitness scenario), the comparable accuracy is .97.

We had expected that CAC functions for 50 and 100 to-be-remembered stimuli would be much different from those in the eyewitness literature. Yet, the surprise is that this strong relationship held even when subjects studied 50 faces and were tested on 100 (half old and half new), and this was done twice. Despite what Tulving and Arbuckle (1966) called *greater input interference* (or *list length* or *cue overload*), the CAC plots in our experiments are rather like those in eyewitness research. High confidence again implies high accuracy. Of course, other sorts of procedures with multiple deceptive lures would alter the relationship between confidence and accuracy (e.g., DeSoto & Roediger, 2014, see also Wixted & Wells, 2017, for unfair lineups). In addition, further experiments to replicate our CAC plots with other types of materials are needed. Theoretical understanding of how and why subjects are so well calibrated in these experiments with large numbers of items must be a task for the future. Given the straightforward nature of CAC analysis (Mickes, 2015) and the fact that the basic procedure was introduced over 20 years ago (Juslin, Olsson, & Winman, 1996), it seems surprising that researchers who study recognition memory have made so little use of this analysis outside the eyewitness identification paradigm. Even its widespread use in eyewitness literature is relatively recent.

A third issue raised by our experiments concerns accuracy for judgments given at the highest value of the confidence scale. Even though the overall CAC is generally the same for the four confidence scales, we asked if different levels of accuracy are associated with the highest confidence rating on each scale (i.e., 4 on a 4-point scale up to 100 on a 100-point scale). Subjects gave many fewer judgments of the highest value as the number of points on the scale increased. For example, in Experiment 1 with word lists, the number of observations involving a confidence rating of 4 when that was the highest value on the scale was 1024; the corresponding values for 5, 20, and 100 for the appropriate scales were 984, 786, and 572. (The same pattern held for Experiment 2; *see* Appendix 2.) Given the decrease in the number of observations, one might expect that accuracy for the highest point would increase with magnitude of the scale. However, the data provided only weak support for this supposition. In Experiment 1, accuracy at the highest values were .87, .93, .92, and .94 for scales using 4, 5, 20, and 100 points, respectively. The comparable values in Experiment 2 with faces were .97, .94, .98, and .98. In each case, there was a marginally significant effect of scale, with the only significant pairwise difference being between the 5- and 100-point values. Once again, in both experiments, a rating of high confidence indicated high accuracy. On the 100-point scale, a rating of 100 indicates an accuracy of nearly 100. Even when subjects give a 4 on a 4-point scale, accuracy is .87 or better. We should note again that this outcome occurred even though we used deceptive lures (lures were primary associates of the target words) in Experiment 1.

These results are consistent with the findings of Mickes et al. (2011), who showed that subjects have trouble scaling high-confidence memories. That is, they discovered that subjects in standard recognition experiments often bunch high-confidence responses at the highest point on the scale, and, when asked to discriminate among those responses, they find it impossible to do so (without feedback). Similarly, in our experiments, subjects bunched responses at the highest points of the scale, and it did not matter too much if the scale provided 4 or 5 ratings or 20 or 100. High confidence means high accuracy.

We examined the relationship of confidence in making correct rejections in our experiments, and we compared CAC plots for correct rejections with those with CAC plots of hits. The different scale types generally produced little or no difference among the proportions of correct rejections in either experiment. In general, the CAC plots are gently positive, with higher confidence leading to higher rates of correct rejections. Correct rejections were considerably lower and less confident in Experiment 1 (with words) than in Experiment 2 (with faces). This could be either because of inherent differences in recognizing words and faces, or because we selected lures for words to be highly associated with the target words, or because of both factors.

One interesting outcome in the direct comparison of CAC plots for hits and correct rejections in both experiments (Fig. 5) is that the CAC function is steeper for hits than for correct rejections. Correct rejections are much more accurate than hits at lower levels of confidence. The opposite occurs at the highest level: Hits display greater accuracy than correct rejections. In other words, subjects seem better calibrated in assessing hits than in assessing correct rejections, and for correct rejections, accuracy of their assessments does not change as much across confidence levels (Palmer, Brewer, Weber, & Nagesh, 2013).

The fact that correct rejections lead to a less well-calibrated relationship between confidence and accuracy is predicted by the standard unequal variance signal detection model of recognition memory (*see* Mickes et al., 2007, 2011). In this model, the target distribution has greater variance than the lure distribution, and Mickes et al. (2007) plotted distributions of confidence ratings for correct rejections and hits that supported this assumption. An interesting property observed in these distributions is bunching at the end points of the scale, especially for hits. That is, subjects make a relatively large number of responses using the highest points on the confidence scale and more for hits than for correct rejections. This pattern can readily be observed in our data by comparing the numbers of observations given at the highest values of the scales in Appendix 1 (hits) with those in Appendix 3 (false alarms). As noted above, Mickes et al. (2011) showed that subjects have great difficulty in scaling the extremely confident old responses for targets, the hits. In discussing their data, Mickes et al. (2011) suggested that a lifetime of experience in evaluating confidence of memories makes the confidence scale natural to use even with no special instructions. People often receive feedback about whether their positive recognition decisions are correct, and they may learn when high confidence means high accuracy. The cases in which high-confidence responses are wrong are ones involving highly similar lures (DeSoto & Roediger, 2014; Roediger & McDermott, 1995), referred to as *deceptive lures* by Koriat (2012). When people make positive recognition judgments, they get feedback (e.g., grades on multiple choice items in education). We suspect it is much harder to gain feedback on events that did not happen (i.e., on “no” decisions to lures). Except on true/false tests, people may not gain much experience on judging how confident they are that an event did not happen. If so, this difference in learning history may account for why the CAC plot for lures is shallower and less well calibrated than that for targets. Of course, these suppositions are speculative and await further empirical examination.

## Conclusions

We obtained little difference in how confidence and accuracy are related across four confidence scales differing widely in magnitude in experiments with both words and faces. Apparently, subjects can readily use these different scales in the same manner. In addition, perhaps because strong memories are hard to scale, subjects providing the highest ratings on the various scales (e.g., 5 on a 5-point scale and 100 on a 100-point scale) show little difference in accuracy. The CAC function for hits is steeper than for correct rejections, in line with standard unequal variance signal detection models. CAC functions provide insight into standard recognition memory experiments with many stimuli, just as they do for eyewitness identification experiments with one event.

## Appendix 1

Number of observations for hits per confidence bin

**Table 2:** Number of observations for hits for experiment 1 (top) and experiment 2 (bottom) per confidence bin at the four-confidence-bin comparison

Scale type	Confidence bins				Total
	1	2	3	4	
Experiment 1					
100-point	229	466	694	1914	3303
20-point	341	504	754	1788	3387
4-point	222	663	755	1803	3443
Experiment 2					
100-point	89	220	298	1174	1781
20-point	67	172	310	1090	1639
4-point	80	240	349	1024	1693

**Table 3:** Number of observations for hits for experiment 1 (top) and experiment 2 (bottom) per confidence bin at the five-confidence-bin comparison

Scale type	Confidence bins					Total
	1	2	3	4	5	
Experiment 1						
100-point	170	311	516	721	1585	3033
20-point	214	362	555	621	1635	3387
5-point	112	326	537	582	1546	3103
Experiment 2						
100-point	79	104	223	377	998	1781
20-point	43	92	177	302	1025	1639
5-point	47	145	221	281	984	1678

## Appendix 2

Number of observations at the most confident point

**Table 4:** Number of observations for hits (top) and correct rejections (bottom) at the most confident point of each scale

Scale type	Experiment 1	Experiment 2
Hits		
100-point	884	572
20-point	963	786
5-point	1546	984
4-point	1803	1024
Correct rejections		
100-point	81	106
20-point	86	340
5-point	350	498
4-point	583	444

## Appendix 3

Number of observations for correct rejections per confidence bins

**Table 5:** Number of Observations for Correct Rejections for Experiment 1 (top) and Experiment 2 (bottom) per Confidence Bin at the Four Confidence Bin Comparison

Scale type	Confidence bins				Total
	1	2	3	4	
Experiment 1					
100-point	381	745	912	983	3021
20-point	686	899	897	592	3074
4-point	468	1183	1004	583	3238
Experiment 2					
100-point	145	553	576	721	1995
20-point	149	451	658	779	2037
4-point	204	595	780	444	2023

**Table 6:** Number of observations for correct rejections for experiment 1 (top) and experiment 2 (bottom) per confidence bin at the five confidence bin comparison

Scale type	Confidence bins					Total
	1	2	3	4	5	
Experiment 1						
100-point	303	447	795	904	572	3021
20-point	386	772	736	780	400	3074
5-point	305	768	1285	775	350	3483
Experiment 2						
100-point	125	293	519	606	452	1995
20-point	77	265	418	611	666	2037
5-point	92	360	513	473	498	1936

## Abbreviations

ANOVA: Analysis of variance
CAC: Confidence-accuracy characteristic
